# Autopsy findings in drivers and passengers from fatal motor vehicle collisions: limited differences in injury patterns and toxicological test results

**DOI:** 10.1007/s12024-021-00359-z

**Published:** 2021-02-20

**Authors:** Jan Mario Breen, Pål Aksel Næss, Christine Gaarder, Arne Stray-Pedersen

**Affiliations:** 1 Division of Laboratory Medicine, Department of Forensic Sciences, Oslo University Hospital, P.O. Box 4950 Nydalen, 0424 Oslo, Norway; 2 Division of Emergencies and Critical Care, Department of Traumatology, Oslo University Hospital, P.O. Box 4950 Nydalen, 0424 Oslo, Norway; 3 Institute of Clinical Medicine, University of Oslo, Oslo, Norway

**Keywords:** Motor vehicle collisions, Autopsy, Injury patterns, Alcohol, Drug, Forensic pathology, Norway

## Abstract

We performed a retrospective study of the injuries and characteristics of occupant fatalities in motor vehicle collisions in southeast Norway. The goal was to provide updated knowledge of injuries sustained in modern vehicles and detect possible differences in injury pattern between drivers and passengers. Forensic autopsy reports, police, and collision investigation reports from 2000 to 2014 were studied, data extracted and analyzed.

A total of 284 drivers, 80 front-seat passengers, and 37 rear-seat passengers were included, of which 67.3% died in front collisions, 13.7% in near-side impacts, 13.5% in rollovers and 5.5% in other/combined collisions. Overall, 80.5% died within one hour after the crash. The presence of fatal injuries to the head, neck, thorax and abdomen were observed in 63.6%, 10.7%, 61.6% and 27.4% respectively. All occupants with severe injuries to the head or neck had signs of direct impact with contact point injuries to the skin or skull. Injuries to the heart and spleen were less common in front-seat passengers compared to drivers. Seat belt abrasions were more common and lower extremity fractures less common in both front-seat and rear-seat passengers compared to drivers. Blood alcohol and/or drug concentrations suggestive of impairment were present in 30% of all occupants, with alcohol more often detected among front-seat passengers compared to drivers.

Few driver-specific and passenger-specific patterns of injury could be identified. When attempting to assess an occupant’s seating position within a vehicle, autopsy findings should be interpreted with caution and only in conjunction with documentation from the crash scene.

## Introduction

Forensic pathologists are relied upon after a fatal motor vehicle collision (MVC) to determine the manner of death, the injury mechanisms following the collision and, if possible, identify the role of each occupant including seating position at the time of the impact. Defining the driver may have key legal implications.

Nevertheless, only a few studies have attempted to distinguish drivers from passengers based on the post-mortem findings [1–4]. Three of these papers are case reports from intensive crash investigations combining postmortem findings with examinations of the vehicle interior and knowledge about occupant movements [1–3]. Except skin bruising from seat belts with opposite patterns, few differences in injury patterns are described. However, Curtin and Langlois reported from a study of nearly 300 MVC fatalities that drivers were more likely to sustain brain injury and skull fractures compared to passengers, whereas splenic injuries were more commonly observed among front-seat passengers [4]. Studies of rear-seat versus front-seat occupants have been performed based on larger crash data sets and have shown that occupants in the rear are more likely to sustain fatal or severe injuries than those in the front seat [5, 6]. However, advances in vehicle safety technologies since the mid-90 s have greatly improved safety for front row occupants, seemingly outperforming the advantages of being seated in the rear [6–8].

Collision-related circumstances such as the consumption of alcohol and/or other psychoactive substances are widely reported for driver fatalities, but have been investigated to a far smaller extent for passengers. Increased knowledge about passenger fatalities may yield important information regarding injury mechanisms and identify high-risk groups.

This explorative autopsy study provides descriptions of occupants and characteristics of the collisions, injuries, safety equipment, circumstances, and toxicology of fatal MVCs in southeast Norway in a 15-year-period. We wanted to explore whether injury patterns differed between front-seat passengers, rear-seat passengers and drivers. The goals were to provide updated knowledge that might assist in determining the seating locations of non-survivors in MVCs and improve the understanding of passenger safety for future preventive strategies.

## Materials and methods

### Data sources

**Fig. 1:**
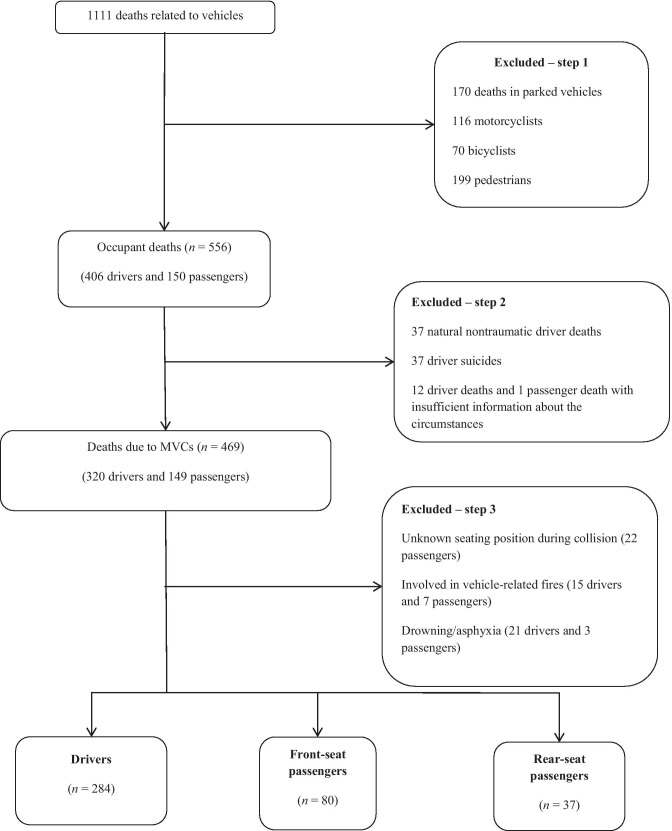
Flow chart of occupant selection and outline of the study

Data on road traffic fatalities between January 2000 and December 2014 were retrospectively collected from the autopsy records at the Department of Forensic Sciences at Oslo University Hospital. The search procedure is previously described in detail [9]. Data from autopsy records were supplemented by reports from the Collision Investigation Team Database at the Norwegian Public Road Administration (NPRA) as well as police reports. For cases prior to 2005, only collision data from police reports were available. All of the forensic autopsies included both internal and external examinations, and sporadically also postmortem CT. Passengers and drivers who died within 30 days after the collision were included. Occupant seating position was obtained from police reports. Nontraumatic deaths and occupants whose seating position was unknown were excluded. An outline of the study is given in Fig. 1.

### Definition of variables

The following information was registered:

- Occupant information: seating position, usage of safety equipment, height, weight, body mass index (BMI), gender, age, injury distribution, postinjury survival time, and toxicology findings.
- Collision-related characteristics: collision type, impact direction, vehicle types involved, vehicle model years, road type, speed limits, traveling speeding, weather conditions, time of day and year, and road visibility.

### Classification of occupant and collision-related variables

In the present study, the term “occupant” referred to the deceased regardless of their location within the vehicle, and so included the driver, front-seat passenger, and rear-seat passengers. Occupant location was categorized into three categories; driver, front-seat passenger and rear-seat passenger. In a subgroup analysis, drivers and front-seat passengers were combined into the group “front-seat row occupants” and compared to rear-seat passengers. Due to the low numbers of passengers in the various rear seating positions, they were all merged into one category for the statistical analyses.

Occupants were categorized as obese, overweight, normal weight, and underweight for BMIs of ≥ 30.0, 25.0–29.9, 18.5–24.9, and < 18.5 kg/m^2^, respectively. Occupants younger than 18 years were not included in the statistical analysis of BMI.

Occupants were considered to have been using a seatbelt if they were restrained by a three-point lap and shoulder belt, or a two-point lap belt, as revealed by the on-site police investigation or if the postmortem examination revealed clear oblique bruising of the chest or horizontal bruising across the abdomen (the so-called seatbelt sign). Detailed data regarding airbag deployment were missing in numerous cases, and most often coded as a dichotomous yes/no variable. Occupants were considered protected by an airbag if airbag deployment was described in reports of the collision scene and no airbag malfunction was recorded. We checked that the vehicle model was indeed equipped with airbags available for the occupant in question in the primary impact direction (front airbags in frontal collisions and side airbags/airbag curtains in side collisions) by using special software (Collision Recovery, version 4.0.3.8). Depending on the level of safety equipment used, occupants were categorized into four restraint groups: (1) seatbelt used and airbag deployed, (2) seatbelt protection only, (3) airbag protection only, and (4) unprotected, which included no seatbelt use and no airbag deployment. There was no information regarding seatbelt misuse, and cases where the use of safety equipment was ambiguous were excluded.

We divided the MVCs into collisions between two vehicles (vehicle–vehicle collisions) and single-vehicle collisions. Collision impacts were categorized into frontal, near-side, rollover, and others (rear, far side, or sideswiped). One impact did not mutually exclude another impact; for example, a collision could involve both a frontal impact and a rollover. We classified the impact type for a particular collision according to which impact that likely contributed most to the fatal outcome, based on available evidence.

The “speeding” category relied on information about the traveling speed being inappropriate (if within the speed limit but too fast during wet/icy/slippery conditions), or about excessive speed (driving above the speed limit), or driving hazardously (e.g. hazardous overtaking or not keeping a sufficient distance from the vehicle in front) as assessed by the NPRA collision analyses. The motor vehicle (MV) types were categorized into sedan/station wagon/hatchback, sport utility vehicle (SUV)/van, lorry/truck/bus, or other (tram/train). Model years were categorized based on industry safety standards and reflecting the introduction of newer occupant protection technology [10], with the MVs grouped in to pre-1994, 1994–1997 (first-generation airbags), 1998–2004 (second-generation, depowered airbags), and 2005–2014 (advanced airbag systems).

### Injury classification

Injuries were classified according to body region, organ system, and injury type (contusion, laceration, hemorrhage, dislocation, or fracture). Injuries considered deadly and the primary cause of death were characterized as “fatal” in the present study. Injury severity assessments were based upon using the Abbreviated Injury Scale (AIS) 2005 manual (Association for the Advancement of Automotive Medicine, 2008) which classifies each body region into the following 6-point ordinal severity scale: 1, minor; 2, moderate; 3, serious; 4, severe; 5, critical; and 6, maximum (currently untreatable). Injuries corresponding to an AIS score of ≥ 3 for internal organs or important blood vessels, and to an AIS score of ≥ 2 in the upper and lower extremities were selected and included in the study.

### Toxicology

The threshold for likely impairment was a blood alcohol concentration (BAC) of ≥ 0.5 g/kg, and for psychoactive medicinal substances or illicit drugs concentrations equivalent to a BAC of ≥ 0.5 g/kg [9].

### Data analyses

Differences in occupant-related and collision-related characteristics as well as in the frequencies of certain injuries between drivers, front-seat passengers, and rear-seat passengers were tested using the chi-square and Fisher’s exact test for binomial categorical variables, and the independent Student’s *t-*test for continuous variables. Univariable and multivariable binary logistic regression was also conducted, using different injuries as outcome variables and adjusting for the following covariates: gender, age, BMI, seating location, safety equipment, impact type, MV type, MV type of collision partner, collision type, high-speed and low-speed roads, speeding, time of day, and season. All statistical analyses were performed using SPSS statistical software (version 22.0, IBM Corporation, Armonk, NY). The conventional significance cutoff level of 5% was used.

## Results

### Demographic data

This study analyzed 401 MVC occupant fatalities: 284 drivers, 80 front-seat passengers, and 37 rear-seat passengers (Fig. 1). The age and gender distributions differed significantly between drivers and passengers (Table 1). There was a clear male predominance in the driver group, whereas both front-seat passengers and rear-seat passengers showed a balanced sex distribution (*p* < 0.001). There were few child fatalities overall, and a higher proportion of elderly (i.e. ≥ 65 years) were observed for front-seat passengers compared with drivers (*p* = 0.032).

**Table 1:** Demographics and collision-related circumstances

	Drivers		Front-seat passengers		Rear-seat passengers		Total	
	(n = 284)		(n = 80)		(n = 37)		(n = 401)	
	*n*	*%*	*n*	%	*n*	%	*n*	%
Gender								
Male	236	83.1	44	55.0	18	48.6	298	74.3
Female	48	16.9	36	45.0*	19	51.4*	103	25.7
Age group								
0–15 years	0	0	3	3.8	4	10.8*	7	1.7
16–24 years	56	19.7	25	31.3*	13	35.1*	94	23.4
25–44 years	111	39.1	22	27.5	8	21.6*	141	35.2
45–64 years	69	24.3	8	10.0	5	13.5	82	20.4
≥ 65 years	48	16.9	22	27.5	7	18.9	77	19.2
BMI category^a^								
< 18.5 kg/m^2^	2	0.7	3	4.2	2	7.7	7	1.8
18.5–24.9 kg/m^2^	106	37.3	28	38.9	13	50.5	147	38.5
25–29.9 kg/m^2^	99	34.9	28	38.9	7	26.9	134	35.1
≥ 30.0 kg/m^2^	77	27.1	13	18.1	4	15.4	94	24.6
Safety equipment^b^								
Unprotected	40	20.2	8	13.1	9	31.0	57	19.8
Airbag only	58	29.3	16	26.2	0	0	74	25.7
Seatbelt only	30	15.2	15	24.6	20	69.0	65	22.6
Seatbelt and airbag combined	70	35.4	22	36.1	0	0	92	31.9
Postcollision survival time								
≤ 1 h	235	82.7	60	75.0	28	75.7	323	80.5
> 1 h and ≤ 24 h	30	10.6	12	15.0	7	18.9	49	12.2
> 24 h and ≤ 1 week	15	5.3	6	7.5	2	5.4	23	5.7
> 1 week and ≤ 2 weeks	2	0.7	1	1.3	0	0	3	0.7
> 2 weeks and ≤ 3 weeks	1	0.4	1	1.3	0	0	2	0.5
> 3 weeks and ≤ 4 weeks	1	0.4	0	0	0	0	1	0.2
Time of day^b^								
0000–0600 h	47	18.5	24	33.8*	6	17.1	77	19.2
0600–1200 h	67	26.4	11	15.5	2	5.7*	80	20.0
1200–1800 h	89	35.0	24	33.8	15	42.9	128	31.9
1800–2400 h	51	20.1	12	16.9	12	34.3	75	18.7
Season								
Winter (December to February)	79	26.8	19	23.8	10	27.0	105	26.2
Spring (March to May)	59	20.8	17	21.3	8	21.6	84	20.9
Summer (June to August)	74	26.1	23	28.8	9	24.3	106	26.4
Autumn (September to November)	75	26.4	21	26.3	10	27.0	106	26.4
Time period								
2000–2004	128	45.1	29	36.3	13	35.1	170	42.4
2005–2009	88	31.0	27	33.8	16	43.2	131	32.7
2010–2014	68	23.9	24	30.0	8	21.6	100	24.9
Road topography^b^								
Curved	115	59.0	47	74.6*	20	76.9	182	64.1
Straight	80	41.0	16	25.4	6	23.1	102	35.9
Road visibility^b^								
Good	173	94.5	57	95.0	22	84.6	252	93.7
Bad	10	5.5	3	5.0	4	15.4	17	6.3
Lighting conditions^b^								
Daylight or road lighting	151	83.0	42	73.7	18	51.4	211	79.6
Twilight or darkness	31	17.0	15	26.3	8	30.8	54	20.4
Speed limit^b^								
High (≥ 70 km/h)	175	75.8	36	58.1	24	82.8	235	73.0
Low (< 70 km/h)	56	24.2	26	41.9*	5	17.7	87	27.0
Speeding^b^								
Yes	51	34.5	26	50.0*	13	52.2	90	40.2
No	97	65.5	26	50.0	11	45.8	134	59.8
MV type^b^								
Sedan/station wagon/hatchback	229	85.8	71	93.4	30	83.3	330	87.1
SUV/van	24	9.0	5	6.6	4	11.1	33	8.7
Lorry/truck/bus	14	5.2	0	0*	2	5.6	16	4.2
MV type of collision partner^b^								
Sedan/station wagon/hatchback	50	27.8	21	60.0*	8	42.1	79	33.8
SUV/van	33	18.3	5	14.3	4	21.1	42	17.9
Lorry/truck/bus	93	51.7	9	25.7*	7	36.8	109	46.6
Tram/train	4	2.2	0	0	0	0	4	1.7
Model year^b^								
Pre-1994	62	34.6	20	33.3	6	24.0	88	33.8
1994–1997	40	22.3	10	16.7	4	16.0	54	20.5
1998–2004	45	25.1	18	30.0	9	36.0	72	27.3
2005–2014	32	17.9	12	20.0	6	24.0	50	18.9
Accident type								
Vehicle–vehicle collision	198	69.7	38	47.5	20	54.1	256	83.8
Single-vehicle collision	86	30.3	42	52.5*	17	45.9	145	36
Impact area								
Frontal	211	74.3	39	48.8*	20	54.1*	270	67.3
Near-side	22	7.7	26	32.5*	7	18.9	55	13.7
Rollover	34	12.0	12	15.0	8	21.6	54	13.5
Others (rear, far-side, or sideswiped)	17	6.0	3	3.8	2	5.4	22	5.5

*Significantly different (*p* < 0.05) compared to drivers (reference group)

^a^Occupants younger than 18 years not included

^b^Information missing/not included in autopsy/police reports in some cases

### Causes of death and injuries

The most common cause of death for both drivers and passengers was injuries to multiple body regions (43.4%, 174/401), followed by isolated injuries to the head (33.7%, 135/401), thorax (19.2%, 77/401), abdomen (2.0%, 8/401), and neck (1.7%, 7/401). Fatal injuries to multiple body regions occurred significantly more often in drivers than in front-seat passengers (47.5% [135/284] vs 28.5% [23/80], *p* = 0.003), but not compared with rear-seat passengers (43.2% [16/37], *p* = 0.623, n.s.).

Table 2 lists the internal injuries detected at autopsy. Front-seat passengers demonstrated significantly lower frequencies of severe injuries to the heart (*p* = 0.001), spleen (*p* = 0.004), and fractures to lower extremities (*p* = 0.037) compared with drivers. Subgroup analysis revealed that front-seat passengers experienced both fewer femur fractures (26.3% [21/80] vs 42.3% [120/284], *p* = 0.009) and leg fractures (13.8% [11/80] vs 27.8% [79/284], *p* = 0.01) compared with drivers. Rear-seat passengers demonstrated a smaller proportion of fractures to the lower extremities (*p* = 0.007), and hinge-fracture to the skull were less common (*p* = 0.048) whereas other skull base fractures occurred more frequently (*p* = 0.002) compared to drivers. Multivariable logistic regression analyses revealed that no significant associations remained after controlling for the covariates.

**Table 2:** Comparison of injury frequencies between occupant groups

	Drivers (n = 284)		Front-seat passengers (n = 80)		Rear-seat passengers (n = 37)		Total (n=401)	
	*n*	*%*	*n*	*%*	*n*	*%*	*n*	*%*
Head injuries								
Any fatal head injury^a^	188	66.2	45	56.3	21	56.8	255	63.6
AIS ≥ 3 injuries:								
Parenchymal brain injury (including brainstem injury)	172	60.6	40	50.0	17	45.9	229	57.1
Intracranial hemorrhage (subarachnoid, subdural, or epidural)	156	54.9	40	50.0	22	59.5	218	54.4
Any type of skull fracture	155	54.6	36	45.0	18	48.6	209	52.1
Skull base – transverse (hinge)	52	18.3	15	18.8	2	5.4*	69	17.2
Skull base – ring	25	8.8	3	3.8	1	2.7	29	7.2
Skull base – other	28	9.9	9	3.8	10	27.0*	47	11.7
Fractures of calvaria/facial skeleton	54	19.0	4	5.0	3	8.1	61	15.2
Crushed skull	40	14.1	8	5.0	3	8.1	53	13.2
Atlanto-occipital dislocation^b^	24	8.5	5	6.3	3	8.1	32	8.0
Vertebral column/spinal injuries								
Any fatal neck injury^a^	34	12.0	5	6.3	4	10.8	43	10.7
AIS ≥ 3 injuries:								
C1-C2 injury^c^	27	9.5	5	6.3	3	8.1	35	8.7
Lower cervical vertebrae fracture^c^	16	5.6	8	10.0	3	8.1	27	6.7
Vertebral artery injury	4	1.4	2	2.5	1	2.7	7	1.7
Thoracic injuries								
Any fatal thoracic injury^a^	178	62.7	45	56.3	24	64.9	247	61.6
AIS ≥ 3 injuries:								
Hemothorax^d^	220	77.5	60	75.0	26	70.3	306	76.3
Multiple rib fractures	207	72.9	53	66.3	22	59.5	282	70.3
Lung contusion, hemorrhage, or laceration	183	64.4	47	58.8	20	54.1	250	62.3
Heart contusion, hemorrhage, or laceration	139	48.9	22	27.5*	13	35.1	174	43.1
Complete or near-complete thoracic aorta laceration	113	39.8	27	33.8	12	32.4	152	37.9
Pericardial laceration	109	38.4	33	27.5	9	24.3	145	34.4
Pneumothorax	85	29.9	24	30.0	13	35.1	122	30.4
Pulmonary vessel laceration	27	9.5	8	10.0	5	13.5	40	10.0
Trachea and main bronchi laceration or rupture	20	7.0	2	2.5	2	5.4	24	6.0
Esophagus rupture	6	2.1	0	0	0	0	6	1.5
Thoracic vertebrae fracture^c^	61	21.5	10	12.5	4	10.8	75	18.7
Abdominal injuries								
Any fatal abdominal injury^a^	79	27.8	18	22.5	13	35.1	110	27.4
AIS ≥ 3 injuries:								
Liver laceration, contusion, or rupture	160	56.3	38	47.5	19	51.4	217	54.1
Intra-abdominal bleeding^d^	128	45.1	29	36.3	19	51.4	176	43.9
Spleen laceration, contusion, or rupture	122	43.0	21	26.3*	21	56.8	163	40.6
Small-bowel (including mesenteric damage) laceration, contusion, or rupture	71	25.0	24	30.0	7	18.9	102	25.4
Colon (including mesenteric damage) laceration or contusion	46	16.2	14	17.5	6	16.2	66	16.5
Diaphragm rupture	45	15.8	11	13.8	5	13.5	61	15.2
Kidney laceration, contusion, or rupture	33	11.6	11	13.8	8	21.6	52	13.0
Pancreas laceration, contusion, or rupture	21	7.4	6	7.5	3	8.1	30	7.5
Urinary bladder laceration, contusion, or rupture	11	3.9	7	8.8	2	8.1	20	5.0
Complete or near-complete abdominal aorta laceration	16	5.6	1	1.3	2	5.4	29	4.7
Stomach laceration, contusion, or rupture	8	2.8	0	0	1	2.7	9	2.2
Lumbar vertebrae fracture^c^	10	3.5	2	2.5	1	2.7	13	3.2
Seatbelt abrasions (“seatbelt sign”) reported	32	11.3	19	23.8*	14	37.8*	65	16.2
Extremity fractures, *AIS ≥ 2*								
Lower extremity	144	50.7	30	37.5*	10	27.0*	184	45.9
Upper extremity	114	40.1	30	37.5	15	40.5	159	39.7
Pelvis	87	30.6	27	33.8	8	21.6	122	30.4

^a^The number of injuries considered to be potentially fatal in that body region

^b^With or without brainstem injury

^c^With or without spinal cord injury

^d^Presence of ≥ 100 ml fresh blood

^*^Significantly different (*p* < 0.05) compared to drivers (reference group)

### Vehicle collision type and injury patterns

In frontal impacts the most common cause of death was injuries to multiple regions (48.5%, 131/270), followed by isolated head injury (27.8%, 75/270), isolated chest injury (19.3%, 52/270), isolated abdominal injury (3.0%, 8/270), and isolated neck injury (1.5%, 4/270). In near-side impacts the most common cause of death was isolated injury to the head (38.2%, 21/55), followed by injuries to multiple regions (34.5%, 19/55), isolated chest injuries (23.6%, 13/55), and isolated neck injuries (3.6%, 2/55). In rollover incidents the most common cause of death was isolated head injuries (61.1%, 33/54), followed by multiple injuries (24.1%, 13/54) and isolated chest injuries (14.8%, 8/54).

In total, 80.5% (323/401) of the occupants had at least one AIS ≥ 3 head and neck injury. In 99.1% of these cases (320/323) contact point injuries to the skin or bone and/or clear contusion injuries to the cerebral cortex indicating direct impact to the head were evident. The three occupants with no signs of blunt trauma to the head died of anoxic brain injury due to positional asphyxia after the MVC.

### Use of seatbelts

Seatbelts were being used by only 50.6% of the drivers at the time of the collision, compared with 60.7% of the front-seat passengers (*p* = 0.165, n.s.) and 69.0% (*p* = 0.063, n.s.) of the rear-seat passengers (Table 1). Information on seatbelt use was available for 71.8% (288/401) of the cases. Seatbelt use was more common among females than among males at the time of the collision (78.9% [60/76] vs 45.8% [97/212], *p* < 0.001).

The age distribution and the frequency of seatbelt use did not significantly differ between drivers, front-seat passengers, and rear-seat passengers. However, all rear-seat passengers older than 55 years were using a seatbelt.

The on-site police investigation revealed that among seatbelt users, only one individual had used a lap belt, the rest had used a three-point lap and shoulder belt. Patterned bruising on the chest or abdominal wall corresponding to the position of the diagonal or horizontal strap of the seatbelt was present in 41.4% of all restrained occupants. This seatbelt sign was more commonly observed for restrained front-seat passengers (51% [19/37] vs 32% [32/100], *p* = 0.037) and rear-seat passengers (70% [14/20] vs 32% [32/100], *p* = 0.001) compared to drivers. When drivers and front-seat passengers were put together to create the group “front-seat row occupants” and then compared to rear-seat row occupants, those seated in the rear-row still more often showed signs of a seatbelt (70% [14/20] vs 37% [51/137], *p* = 0.005). We found no associations between a seatbelt sign and the injury pattern or cause of death.

### Alcohol and/or drug impairment

Blood alcohol and/or drug concentrations indicative of impairment at the time of the collision were present in 30.3% of the population (Table 3). Among front-seat passengers with concentrations suggestive of impairment, most were males (70.0%, 21/30), aged 16–44 years (90.0%, 27/30) and involved in a collision during evening/nighttime hours (78%, 21/27). Seatbelt use was observed by 44% (12/27) of these front-seat passengers compared to 23% (12/52) of the impaired drivers (*p* = 0.05, n.s.). Impairment by alcohol alone was found among 30.8% of the front-seat passengers, with the prevalence being significantly lower in drivers (Table 3). All of the six rear-seat passengers with alcohol/drugs in their blood were also males and aged 16–44 years.

**Table 3:** Occupants impaired by psychoactive substances

	Drivers (n = 283)^a^		Front-seat passengers (n = 78)^a^		Rear-seat passengers (n = 35)^a^		Total (n = 396)	
	*n*	*%*	*n*	*%*	*n*	*%*	*n*	*%*
Any substance above study threshold levels	84	29.7	30	38.5	6	17.1	120	30.3
Alcohol (> 0.5 g/kg)	47	16.6	24	30.8*	4	11.4	75	19.0
Psychoactive medicinal drugs	32	11.3	5	6.4	2	5.7	39	9.8
Illicit drugs	33	11.7	10	12.8	2	5.7	45	11.4
Mixed alcohol/medicinal/illicit drugs	26	9.2	9	11.5	2	5.7	37	9.3

^*^Significantly different (*p* = 0.006) compared to drivers (reference group)

^a^Toxicology information was missing in 5 cases; 1 driver, 2 front-seat passengers, and 2 rear-seat passengers

Compared to sober occupants, drivers and passengers with drugs/alcohol in the blood were associated with being in MVs that were driving above the speed limits/driving too fast for the road conditions/driving hazardously at the time of the collision (65.8% [48/73] vs 27.5% [41/149], *p* < 0.001). They were also more often colliding during evening/nighttime hours (48.3% [71/147] vs 18.4% [38/206], *p* < 0.001) and involved in rollover incidents (25.0% [30/120] vs 8.4% [23/274], *p* < 0.001). Alcohol or drug impaired individuals were more commonly involved in single-vehicle collisions (62.5% [75/120] vs 24.5% [67/274], *p* < 0.001) and colliding in a curved road topography (77.7% [73/94] vs 57.4% [108/188], *p* = 0.001).

## Discussion

This study provides a summary of the injuries sustained by occupants who died in MVCs in southeast Norway during a 15-year-period. The main finding is that only minor differences in injury patterns were observed between drivers, front-seat and rear-seat passengers despite variations in gender and age distribution as well as other collision-related characteristics. At an individual level, the details and tissue changes of a lesion can indeed depict the injury mechanism. Injuries caused by primary impacts with the interior of the vehicle may have individual features that differ from injuries caused by secondary impacts from loose luggage or unrestrained fellow occupants. Bruising and fractures can be matched with contact points of the interior of the vehicle for comparison and thus assessment of the injury mechanism. This however, necessitates access to data from a thorough investigation of the crash scene and the vehicles involved. In daily practice, such data are often lacking. On the other hand, in larger databases, injuries are categorized according to severity and less severe contact point bruising injuries are rarely reported. Even when only studying a single type of collision, e. g. frontal impacts, there is still a large potential for variation in mechanism of injury dependent upon factors such as the force of the impact, the structure of the vehicles involved, the position and the anthropometry of the occupant, as well as use and misuse of restraints and differences in the safety equipment of the vehicle. Systematic comparison of the injury patterns in drivers, front-seat passengers, and rear-seat passengers involved in MVCs with modern vehicles are scarce in the literature.

In our dataset, driver fatalities were more frequently the result of front collisions with a heavier collision partner, whereas a significantly higher proportion of front-seat passengers were killed in near-side impact, often on curved roads. Despite these variations very few differences in injuries were observed. A higher occurrence of injuries to the heart among drivers may be explained by both differences in the vehicle interior (steering wheel) and collision type (high-energy front collisions). Curtin and Langlois reported that splenic injuries were more commonly observed among front-seat passengers compared to drivers [4]. We observed the opposite, that splenic injuries were less common for front-seat passengers. A likely explanation is that the spleen is more vulnerable to impacts and door intrusions from the left side. In Norway the front passenger seat is on the right side of the vehicle, whereas in Australia it is located on the left side. Drivers had higher rates of lower extremity fractures than both front-seat and rear-seat passengers. A possible explanation is that frontal impacts most often involve the driver’s side of the front, making drivers more prone to dashboard and floor-pan intrusion and foot entrapment by the pedals [11].

The seatbelt sign, more precisely the marks from the oblique part of the seat belt towards one of the shoulders, can help differentiate occupant locations within the MV, but this sign was only present in 41.4% of the cases. Absence of a seatbelt sign does not necessarily indicate that no seatbelt was used [12].The overall seatbelt use rate was low (only 50–60%) for drivers and front-seat passengers. It is well established that seatbelts and airbags are effective protective devices for reducing morbidity and mortality in MVCs [13]. It is worth mentioning that all rear-seat passengers older than 55 years in the present study were using a seatbelt. Reduced physical capabilities combined with frailty mean that the risks of injury and death are higher in older than younger occupants [5, 14]. However, a higher proportion of seatbelt use in the rear-seats among younger occupants might have prevented deadly injuries for some young individuals. Therefore, MVCs involving young passengers both in the front-seat and rear-seat seem to share many characteristics to those involving young drivers.

Near-side impacts may be more dangerous than far-side impacts as an occupant is exposed to direct contact with the intruding side structure of the vehicle [15, 16]. Compartment intrusion and the narrowing of the space available to occupants cause lateral tilting of the head and neck, and also impact trauma to the chest. Seatbelts provide little protection against these types of injury [15, 16]. Side-impact airbag protection systems in modern vehicles have shown to reduce the risk of head and thoracic injuries in near-side impacts [16, 17]. However, very few of the vehicles involved in the present study were equipped with side airbags since more than half of them were manufactured before 1997 and side airbags were not introduced until the late 1990s [18]. Older vehicles are typically over-represented in MVC fatalities, indirectly demonstrating the significant improvements made in passive safety equipment for occupants in modern vehicles [19].

Severe (AIS ≥ 3) head and/or neck injuries were observed in 80.5% of the occupants. They all had signs of impact points on to the skin, soft tissue and/or bone of the head/upper neck. It has been assumed that severe injures may occur due to pure inertial loading of the head without impact, by a whiplash mechanism involving abrupt jerking movement of the head in a backward or forward direction. Whiplash injuries are very common among survivors of MVCs, however, the term is poorly defined and describes all types of neck strains and sprains [20]. In the majority of cases, there is a lack of an organic base for the symptoms experienced by the occupants, but it has been argued that some experience injuries to the neck ligaments, the facet joints and intervertebral discs of the neck vertebrae [20]. Autopsy studies with detailed dissection of the cervical spine have detected facet joint injuries, but only when concomitant presence of skull fractures or other injuries implying direct impact to the head [21]. There are scattered case reports of major injuries to the neck without signs of blunt direct trauma contact points [22, 23], but such contact points may be easily missed unless a thorough examination of both the skin and soft tissue is performed and the validity of such proposed mechanisms has been questioned earlier [24]. The present study suggests that fatal injuries to the head and neck from MVC in modern vehicles consistently result from a trauma with some type of direct impact. Hyperextension, hyperflexion and rotational shearing forces are likely part of the injury mechanism, but nevertheless an impact seems to be obligatory.

Earlier research suggested that the rear-seat row was safer for occupants in crashes than the front-seat row [25, 26]. However, recent studies of restrained rear-seat occupants now point to a higher risk of death and severe injury than for belted front-seat occupants [5–8]. MVs from between 1997 and 2007 [7], as well as from 2007 and onwards [5] demonstrate lower protection in the rear-seat versus the front-seat, likely attributable to improvements in front-seat occupant protection (i.e., incorporation of load limiters and pre-tensioners, increased vehicle stiffness, and airbag availability) [5, 8]. Compared to front-seat occupants (drivers and passengers), the rear-seat passengers have been shown to be more likely to sustain chest injuries, primarily caused by seatbelt loading [8]. This finding was not reproduced in our dataset. However, the number of included rear-seat passengers in our study is low; hence the statistical power is poor. Yet, seatbelt marks were clearly more evident among rear-seat passengers than front-seat row occupants perhaps pointing to a heavier seatbelt loading in the rear row.

Previous studies indicate that the presence of a passenger increases the crash risk and is associated with a worse outcome [27, 28]. Having passengers—particularly teenage and multiple passengers—can affect the driving behavior and the risk of fatal collisions, and young drivers are more likely to collide while carrying passengers [29, 30]. We found that 38.5% of the front-seat passengers were likely to be influenced by alcohol and/or drugs at the time of the collision. Most of these were in the age range 16–44 years, and had collided during evening/nighttime hours, not using a seatbelt at the time of the collision. The current literature indicates that neither drivers nor passengers make safe decisions about using vehicles after substance use. There is evidence that passengers influence the decisions and risk-taking behaviors of drivers by distracting the driver or by promoting risky behaviors such as speeding, in particular if the passengers have consumed alcohol or other drugs [30, 31]. Passengers and drivers tend to share similar drinking behaviors [32, 33]. Drinking passengers constitute a high-risk group that is often not considered in prevention efforts [34].

## Limitations

In a retrospective study of fatalities, the lack of an appropriate control group leads to selection bias. Since survivors were not included, the protective effect of restraining and airbag devices is precluded. The circumstances of the collision were obtained from police reports rather than from thorough collision reconstructions, with limited data available on actual impact forces, use and positioning of seatbelts and airbag function. The kinetic energy in MVCs involving newer vehicles equipped with modern safety equipment was likely higher than in crashes with older vehicles, confounding comparison of injury patterns. We were unable to match occupants seated in the same vehicle, which made it even harder to control for confounding collision-related characteristics such as the vehicle speed and vehicle model [35], or whether drivers and passengers shared a drinking association.

As expected, there were noticeable variations in the level of the detail of injury descriptions in which some pathologists had described “multiple contusion or laceration” in an internal organ while others reported the exact depth and length of each laceration. These discrepancies made it difficult to exactly quantify the injuries within a particular region or organ. Cutaneous and skeletal injuries corresponding to an AIS score of < 2 and sidedness of such injuries were excluded. In hindsight, this should have been incorporated in our analyses.

## Conclusion

This study presents a detailed list of the injuries and characteristics of MVC fatalities on Norwegian roads. Only minor differences in injury patterns between drivers and passengers were revealed. In terms of the practical daily work of the forensic pathologist, autopsy findings must be interpreted in conjunction with a thorough photographs-based documentation of the crash scene and the circumstances of the collision, before attempting to make any assumptions regarding the seating position of the victim at the time of the crash.

Front-seat passenger fatalities were associated with factors indicative of low energy crashes (e.g. near-side impacts, single-vehicle collisions, low weight crash partner), sustaining injuries to multiple body regions less often than drivers. The proportion of subjects with blood alcohol levels exceeding 0.5 g/kg was higher for front-seat passengers than for drivers. These issues should receive more attention in future road safety campaigns.

## Key points

1. Despite dissimilarities in crash characteristics as well as age and sex distribution, only minor differences in injury patterns were observed between driver and passenger fatalities.
2. Front-seat passengers were overrepresented in near-side impact collisions and had fewer injuries to the heart, spleen and extremities than drivers.
3. Alcohol levels exceeding 0.5 g/kg were more often detected in front-seat passengers than in drivers.
4. Severe AIS ≥ 3 injuries to the head or neck due to direct impact to the head during the crash were observed in 80.5 percent of all occupants.
5. Attempting to identify an occupant’s seating position based solely on autopsy results is not advised.

## Data Availability

The data will not be deposited.
